# Plasma Treatment Limits Cutaneous Squamous Cell Carcinoma Development In Vitro and In Vivo

**DOI:** 10.3390/cancers12071993

**Published:** 2020-07-21

**Authors:** Gabriella Pasqual-Melo, Thiago Nascimento, Larissa Juliani Sanches, Fernanda Paschoal Blegniski, Julya Karen Bianchi, Sanjeev Kumar Sagwal, Julia Berner, Anke Schmidt, Steffen Emmert, Klaus-Dieter Weltmann, Thomas von Woedtke, Rajesh Kumar Gandhirajan, Alessandra Lourenço Cecchini, Sander Bekeschus

**Affiliations:** 1 ZIK *plasmatis*, Leibniz Institute for Plasma Science and Technology (INP), Felix-Hausdorff-Str. 2, 17489 Greifswald, Germany; gabriella.pasqual-melo@inp-greifswald.de (G.P.-M.); sanjeev.sagwal@inp-greifswald.de (S.K.S.); julia.berner@inp-greifswald.de (J.B.); anke.schmidt@inp-greifswald.de (A.S.); weltmann@inp-greifswald.de (K.-D.W.); woedtke@inp-greifswald.de (T.v.W.); rajesh.gandhirajan@inp-greifswald.de (R.K.J.); 2 Department of General Pathology, State University of Londrina, Rodovia Celso Garcia Cid, Londrina 86051-990, Brazil; thiagohenrique_d_n@hotmail.com (T.N.); larisanches_89@hotmail.com (L.J.S.); fpblegniski@gmail.com (F.P.B.); julya.bianchi@gmail.com (J.K.B.); alcecchini@uel.br (A.L.C.); 3 Clinic for Oral, Maxillofacial, and Plastic Surgery, Greifswald University Medical Center, Sauerbruchstr., 17475 Greifswald, Germany; 4 Clinic for Dermatology and Venereology, Rostock University Medical Center, Strempelstr. 13, 18057 Rostock, Germany; steffen.Emmert@med.uni-rostock.de; 5 Institute for Hygiene and Environmental Medicine, Greifswald University Medical Center, Walther-Rathenau-Str. 48, 17489 Greifswald, Germany

**Keywords:** kINPen, plasma medicine, plasma sources, reactive oxygen and nitrogen species, cutaneous squamous cell carcinoma (SCC)

## Abstract

Cutaneous squamous cell carcinoma (SCC) is the most prevalent cancer worldwide, increasing the cost of healthcare services and with a high rate of morbidity. Its etiology is linked to chronic ultraviolet (UV) exposure that leads to malignant transformation of keratinocytes. Invasive growth and metastasis are severe consequences of this process. Therapy-resistant and highly aggressive SCC is frequently fatal, exemplifying the need for novel treatment strategies. Cold physical plasma is a partially ionized gas, expelling therapeutic doses of reactive oxygen and nitrogen species that were investigated for their anticancer capacity against SCC in vitro and SCC-like lesions in vivo. Using the kINPen argon plasma jet, a selective growth-reducing action of plasma treatment was identified in two SCC cell lines in 2D and 3D cultures. In vivo, plasma treatment limited the progression of UVB-induced SSC-like skin lesions and dermal degeneration without compromising lesional or non-lesional skin. In lesional tissue, this was associated with a decrease in cell proliferation and the antioxidant transcription factor Nrf2 following plasma treatment, while catalase expression was increased. Analysis of skin adjacent to the lesions and determination of global antioxidant parameters confirmed the local but not systemic action of the plasma anticancer therapy in vivo.

## 1. Introduction

Non-melanocytic skin cancer (NMSC) is one of the most frequently diagnosed types of cancer [1]. It includes, for instance, basal cell carcinoma (BCC) and squamous cell carcinoma (SCC), which represent up to 20% of all skin cancers [2]. Recent work using genetic mouse models of BCC described that stem and progenitor cells are the probable sources of BCC initiation [3]. This type of NMSC can be locally destructive, but metastasis is rare [4]. SCC is more aggressive and characterized by an uncontrolled proliferation of squamous cells in the epidermal layer. Invasive SCC shows masses of atypical keratinocytes proliferating into the dermis. SCCs often contain squamous pearls, a keratinized structure, wherein abnormal squamous cells form concentric layers and occasionally mitotic figures [5]. Frequent exposure to ultraviolet (UV) light is described as an inducer of NMSCs. Especially, UVB exposure is known to be the principal carcinogenic agent that causes mutations in the DNA of keratinocytes, leading to the development of SCC [5], the so-called multistep photocarcinogenesis. For the SCC, intermittent sun exposure in the early decades is also part of its etiology [6]. A more detailed view of SCC etiology, pathogenesis, and treatment options has been outlined previously [7,8,9]. Here, we tested a UVB irradiation protocol, which led the development of SCC in nude mice.

Cold physical plasma is a highly energetic, partially ionized gas that can be operated at body temperatures [10]. One of the major biologically active components of medically relevant cold physical plasma devices is reactive oxygen and nitrogen species (ROS/RNS) formed through reactions with molecules present in the ambient air. The range of applications for this technology mainly includes dermatology and skin-based infections, oncology, and dentistry, among others [11,12,13]. Plasma treatment of non-healing wounds and ulcers is the most established clinical application to date, as evidenced by several approved medical plasma devices in Europe [14]. Oncology is an emerging topic in plasma medicine, with positive results achieved so far in preclinical research on skin cancer [15,16,17]. Several palliative tumor patients have already benefited from plasma therapy [18,19]. In contrast to chemotherapy and radiotherapy, a feature of medical plasma applications is the lack of side effects observed so far in the preclinical [20,21,22] and clinical setting [23,24,25]. Another attractive feature of plasma treatment is a certain degree of selectivity for killing cancer cells, predominantly via apoptosis [26,27,28,29]. More detailed views on plasmas for cancer treatment have been reviewed recently [30,31,32].

Several promising targeted agents for SCC treatment exist, including epithelial growth factor receptor (EGFR) antagonists, small-molecule tyrosine kinase inhibitors (TKIs), proteasome inhibitors, and anti-PD-1/PD-L1 antibodies [5]. However, these therapies come at high costs and often with severe side-effects, motivating the development of novel antitumor approaches and technologies targeting SCC or preventing its progression from lesions. In the present study, we tested cold physical plasma as an in-this-field novel agent for tackling skin squamous cell carcinoma in vitro and its development and progression in vivo.

## 2. Results

### 2.1. Plasma Treatment Was Selectively Cytotoxic to SCC Cells In Vitro

**Figure 1:**
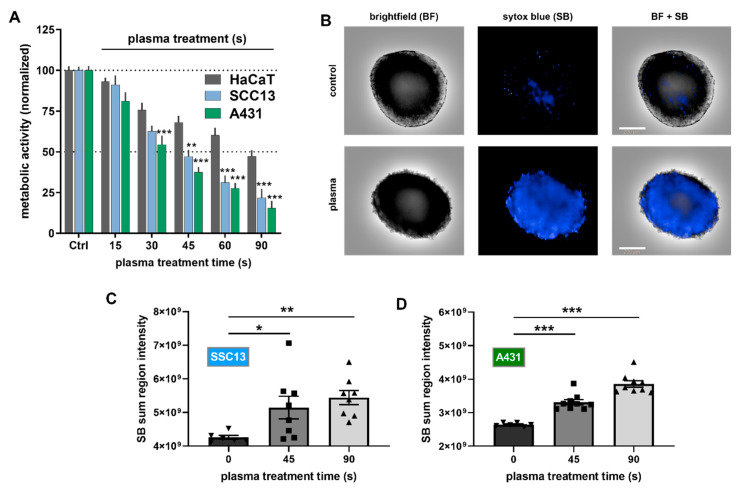
Plasma treatment of SCC in vitro. (**A**) Metabolic activity of malignant squamous cell carcinoma (SCC) (A431, SCC13) and non-malignant HaCaT keratinocytes 24 h post-exposure to plasma; (**B**) representative images of 3D tumor spheroids with and without plasma treatment (90s); (**C**,**D**) quantitative sytox blue (SB) analysis in SCC13 (**C**) and A431 (**D**) tumor spheroids 24 h post plasma treatment. Results are expressed as the mean ± SEM, from 2 to 3 experiments with several replicates each. Statistical analysis was performed using two-way, (**A**) against HaCaT keratinocytes, or one-way (**C**,**D**) analysis of variances against the control condition with *p* < 0.05 (*), *p* < 0.01 (**), and *p* < 0.001 (***). Scale bar = 200 µm.

To analyze the cytotoxicity of plasma treatment in 2D cultured cells in vitro, the metabolic activity of SCC cells (A431, SSC13) and HaCaT keratinocytes were analyzed 24 h after plasma exposure (Figure 1A). A treatment time-dependent decline was observed, and plasma treatment was significantly more cytotoxic in malignant keratinocytes (A431, SCC13) compared to non-malignant keratinocytes (HaCaT). Next, the cytotoxicity of plasma was investigated in 3D multicellular spheroids using high content imaging microscopy (Figure 1B). HaCaT keratinocytes do not form 3D spheroids, which is why they could not be studied using this assay. Plasma treatment showed a treatment time-dependent cytotoxicity in SCC13 (Figure 1C) and A431 (Figure 1D) cells.

### 2.2. Growth of UVB-Induced Skin Lesions Was Limited by Plasma Treatment

**Figure 2:**
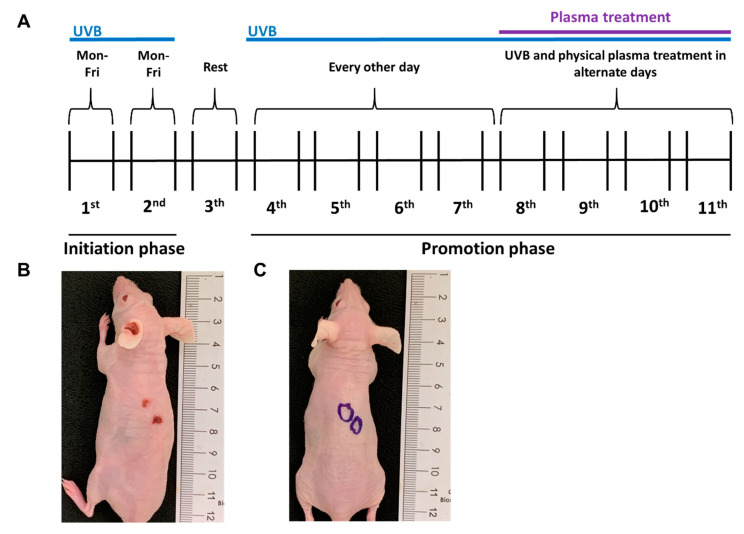
Animal model of UVB-induced skin lesions. (**A**) Timeline of the experiment; (**B**) representative animal of the UVB group right before the first plasma treatment (at the end of the seventh week); (**C**) representative animal of the plasma treatment group (without UVB-induced lesions, at the end of the 11th week after several plasma treatments); the circles indicate the location of the plasma treatment.

**Figure 3:**
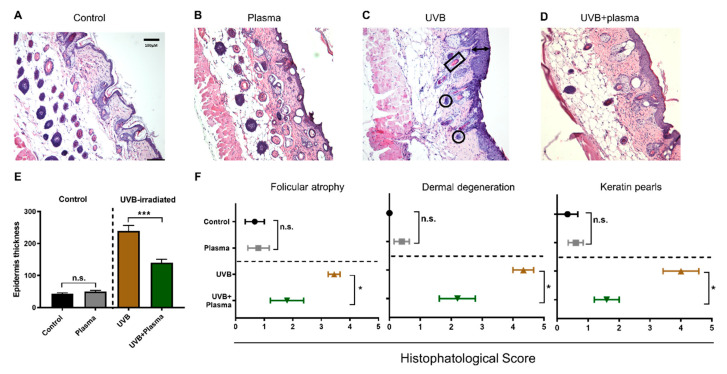
Histopathological evaluation. (**A**–**D**) Representative images of murine skin sections stained with hematoxylin and eosin (H&E) from the untreated (control) (**A**), plasma (**B**), UVB (**C**), and UVB + plasma (**D**) treated mice, respectively (40×); (**E**) quantification of the epidermal thickness (see black arrow in C as an example); (**F**) histopathological score of characteristics related to SCC, which includes follicular atrophy, dermal degeneration, and keratin pearls (see black square in C as an example) [33]. The black circles (**C**) indicate nests of dysplastic squamous cells reaching the dermis. Results are expressed as the mean ± SEM from 4 to 5 animals per group. Statistical analysis was performed using an unpaired t-test with *p* < 0.05 (*) and *p* < 0.001 (***). Scale bar = 100 µm; n.s. = not significant.

The next question was whether plasma treatment also showed efficacy against the progression of UVB-induced skin lesions in vivo. For this, a syngeneic model in nude, immunocompetent mice was used. UVB light was used to induce skin lesions with features of cutaneous SCCs in these animals. After 7 weeks of UVB radiation, according to our protocol (Figure 2A), the animals developed skin lesions comparable with histopathological characteristics of SCC (Figure 2B). In the following weeks, the skin lesions were subjected to plasma treatment in alternated days with continued UVB irradiation to see whether plasma treatment alters the development of the disease (Figure 2C). After 4 weeks of plasma treatment, the animals were euthanized and the skin lesions and their progression status were analyzed via histopathology, according to standardized parameters, as previously described [33]. Unlike the control groups (Figure 3A,B), the UVB irradiation induced an increase of epidermal thickness (Figure 3C,E). Plasma treatment controlled this growth (Figure 3D,E). Blinded histopathological scoring was used to evaluate several characteristics of putative carcinogenic transformation. It was found that plasma treatment significantly reduced the degree of follicular atrophy, dermal degeneration, and keratin pearls induced by the UVB treatment (Figure 3F). At the same time, repeated plasma treatment of the skin of mice not subjected to UVB treatment did not induce pathological or pre-malignant alterations of the skin, as evidenced by the lack of epidermal thickening, follicular atrophy, dermal degeneration, and keratin pearls (Figure 3E,F). These results suggested that plasma treatment was not only safe to be applied onto the skin but also was able to control the growth and consequences of malignant keratinocytes growth in vivo.

### 2.3. Plasma Treatment Decreased Proliferation in UVB-Induced Skin Lesions

**Figure 4:**
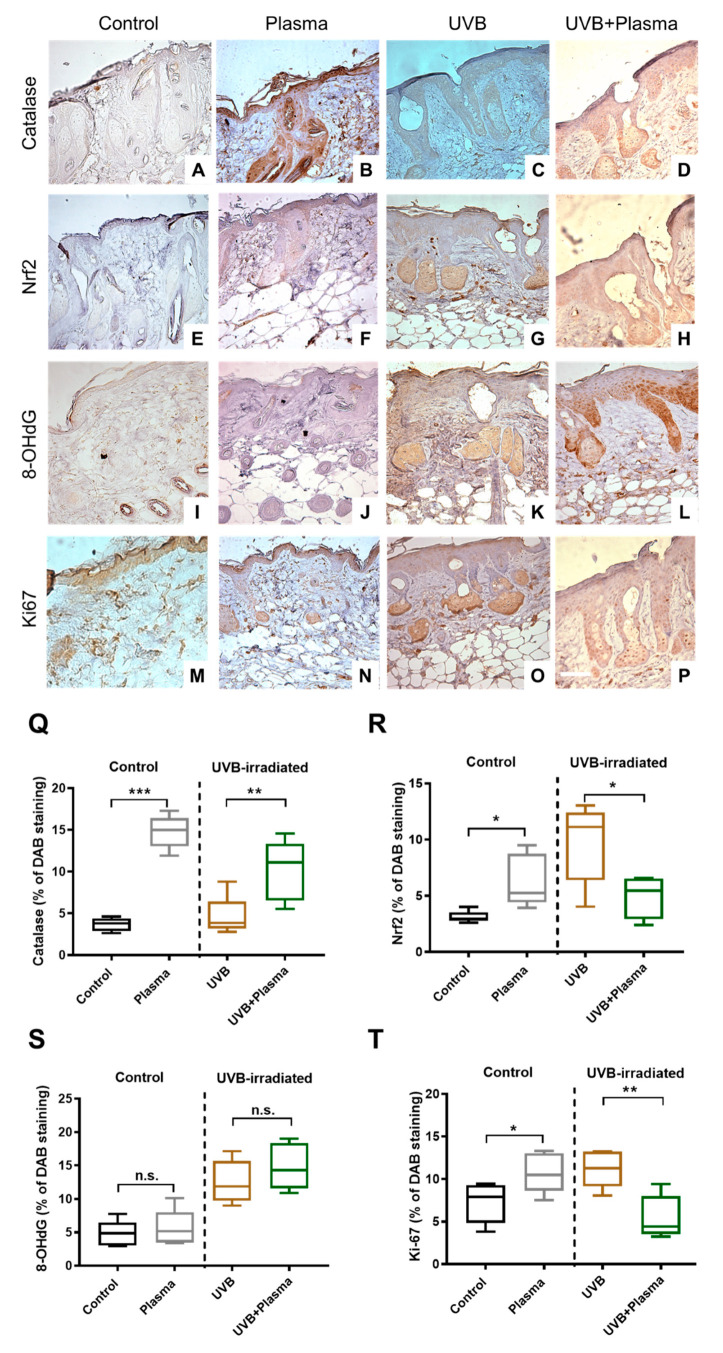
Immunohistochemistry. (**A**–**P**) Representative tissue sections stained with antibodies targeting catalase (**A**–**D**), nuclear factor E2-related factor 2 (Nrf2) (**E**–**H**), 8-hydroxy-2′—deoxyguanosine (8-OHdG) (**I**–**L**), and Ki-67 (**M**–**P**) in control, plasma, UVB, and UVB + plasma treated mice (100×); (**Q**–**T**) quantification of 3,3′-diaminobenzidine (DAB) staining. Results are expressed as boxplots from 4 to 5 animals per group. Statistical analysis was performed using an unpaired t-test with *p* < 0.05 (*), *p* < 0.01 (**), and *p* < 0.001 (***). Scale bar = 200 µm; n.s. = not significant.

To analyze putative mechanisms related to the effects observed, immunohistochemical analysis of the UVB-induced skin lesions was performed. Since plasma treatment generates a plethora of reactive oxygen and nitrogen species simultaneously, which subsequently act on the skin, it was natural to investigate parameters of the antioxidant defense. Plasma treatment induced an increase of catalase in both non-UVB-treated skin and UVB-treated skin (Figure 4A–D and Q). This demonstrated that plasma treatment was recognized as a therapeutic agent in skin cells, leading to an increased translation of the antioxidant enzyme catalase. Catalase expression is under the control of nuclear factor E2-related factor 2 (Nrf2) [34], an essential antioxidant transcription factor. Accordingly, the tissues were stained for Nrf2 expression (Figure 4E–H and R), and its significant upregulation in UVB-challenged skin of mice was found (Figure 4G). In contrast, plasma treatment significantly reduced Nrf2 levels in UVB-induced skin lesions (Figure 4H), suggesting alternative pathways increasing catalase expression in this condition. However, Nrf2 levels were markedly elevated in UVB-treated skin compared to non-irradiated skin, indicating a putative involvement of Nrf2 in the pathology of SCC. Since plasma generates a wealth of free radicals that were previously linked to genotoxic activity, the next question was whether plasma treatment increased the presence of oxidative DNA products as assayed using 8-hydroxy-2′—deoxyguanosine (8-OHdG) antibodies. As expected, the UVB-induced SSC lesions showed a substantial elevation of 8-OHdG staining when compared to unirradiated skin (Figure I–L and S). Plasma treatment was observed to elevate the levels of 8-OHdG significantly; however, it was not observed in non-UVB-irradiated skin (Figure 4I,J) or UVB-induced skin lesions (Figure 4K,L). This supports previous reports that a direct genotoxic effect of plasma-derived reactive species in living cells is unlikely [20,21,35]. Finally, tissue staining of the cell proliferation marker Ki67 was performed (Figure 4M–P and T). Interestingly, plasma treatment of non-irradiated, healthy skin significantly promoted the proliferation of cells in this tissue (Figure 4M,N). This is in line with the wound-healing promoting properties reported for the kINPen plasma therapy [36]. By contrast, plasma treatment of UVB-induced skin lesions significantly decreased the pathological proliferation of this cancerous condition (Figure 4O,P). Altogether, our data confirm not only the stimulating properties of plasma therapy in healthy skin but also the growth-controlling effects of plasma treatment in malignant skin disease.

### 2.4. Analysis of Remote Skin and Systemic Antioxidants Confirmed the Local Activity of Plasma

**Figure 5:**
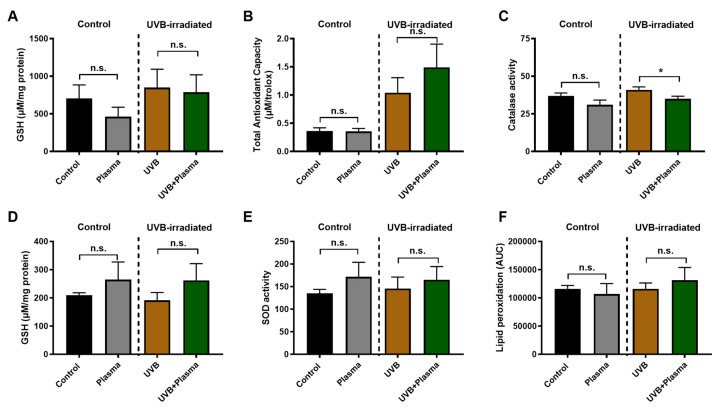
Systemic antioxidants and analysis of non-lesional, adjacent skin. (**A**–**C**) Analysis of adjacent skin for GSH content (**A**), total-trapping antioxidant capacity (**B**), and catalase activity (**C**); (**C**–**F**) analysis of systemic antioxidants in red cells of GSH (**D**), superoxide dismutase (SOD) (**E**), and lipid peroxidation (**F**). Results are expressed as boxplots from 3 to 5 animals per group. Statistical analysis was performed using an unpaired t-test with *p* < 0.05 (*); n.s. = not significant.

It is known, and further evidence was provided in this work, that plasma treatment has profound effects, seen locally at the site of treatment [24,37,38,39,40]. It is often assumed that such effects do not spread regionally or systemically, but direct evidence of this assumption is scarce. Skin that was adjacent (several millimeters apart) to the plasma treatment sites was therefore investigated for several antioxidant parameters. This was done in both mice with UVB-induced skin lesions and non-irradiated mice. Analysis of the major antioxidant glutathione (GSH, Figure 5A), as well as the total antioxidant capacity as determined by total radical trapping antioxidant parameter (TRAP) assay (Figure 5B), did not show any significant difference between animals subjected to plasma treatment and non-irradiated controls. However, when analyzing the amount of active catalase, a small but significant decrease was observed in the UVB group (Figure 5C). Next, a potentially systemic action of plasma treatment was investigated by analyzing erythrocytes. The amount of GSH (Figure 5D), superoxide dismutase (SOD) activity (Figure 5E), and lipid peroxidation (malondialdehyde (MDA), Figure 5F) in blood plasma was not significantly different between the plasma-treated and non plasma-treated animals. This indicates that the activity of plasma-derived reactive species is mainly limited to the treatment site, potentially with small regional but not systemic effects of the exposure.

## 3. Discussion

SCC is characterized by an atypical proliferation of invasive squamous cells under extensive progression [41]. Although the vast majority of SCC can be treated with local or surgical destructive methods, a more aggressive subgroup can metastasize and induce significant morbidity and mortality [42]. Furthermore, large recurrent tumors, deeper than 2 mm, undifferentiated cells, and invading perineural or lymphovascular structures are some characteristics of a more aggressive SCC [43]. Several risk factors have been described for the development of SCC, but exposure to UVB irradiation for many decades that stands out as the most harmful [44]. Among the UV spectrum, UVB has higher energy levels than UVA and typically damages the outermost layers of the skin, interacting directly and indirectly with DNA [45], eventually causing skin cancer [33]. Our study showed that the animals developed a macroscopic skin lesion with histopathological characteristics similar to those of SCC after 7 weeks of UVB-irradiation. Plasma treatment was used as a therapeutic intervention, which limited cell proliferation in the lesions.

For SCC, surgery is the treatment of choice, and radiotherapy is performed when the cancer is inoperable. However, both lead to ablation, not only of the tumor but of a large perimeter of the skin, not to mention the side effects caused by radiotherapy [46]. A therapeutic alternative that doesn’t not lead to mutilations and deformities is required. Several reports have noted a decline in SCC growth after plasma treatment. In a xenograft model in immunodeficient mice, human SCC cells were injected subcutaneously and treated with plasma daily [47]. Besides a reduction of tumor growth, an increase in caspase-3 and apoptosis, as well as NADPH-oxidase 3 (NOX3), was observed in the plasma-treated tumor tissue. NOX3 activity is restrained by toll-like-receptor 4 (TLR4) signaling [48], and a higher expression or activation of NOX3 results in enhanced superoxide production, which rapidly dismutates to hydrogen peroxide (H_2_O_2_) [49]. Although NOX3 expression was not investigated in the present work, it is possible that increased endogenous ROS production may have contributed to increasing catalase expression observed in our model, an enzyme that scavenges H_2_O_2_ [50]. A study that subcutaneously injected syngeneic murine SCC7 cells into C3H/HeJ mice showed a reduction of tumor growth after daily treatment with plasma for 1 week [51]. The authors found an increase in mitochondrial E3 ubiquitin-protein ligase 1 (MUL1) levels, a protein responsible for the degradation of, e.g., the pro-oncogenic kinase, AKT (protein kinase B). The results were confirmed in a xenograft SCC animal model in the same study, giving evidence that plasma treatment is capable of reducing cell growth of injected SCC cells in vivo. However, we show here for the first time that plasma treatment also limits endogenously generated, UVB-induced skin lesions with characteristics of SCC. As our model is per definitionem syngeneic, it is conceivable in this regard that cells of the immune system also might have contributed to the effects observed, as some reports in the literature have suggested plasma cancer treatment for skin cancer-bearing mice [15,16,52,53].

In our study, the effects of plasma treatment on healthy skin not subjected to UVB radiation and subsequent induction of pathological skin characteristics was tested. Plasma treatment did not induce increased epidermal thickening, follicular atrophy, dermal degeneration, or keratin pearls, all being a hallmark of SCC in this mouse model and appearing with constant UVB irradiation [54]. This absence of tumorigenic potential was also previously shown in our work using SKH1 mice and investigation of tissues 1 year after repetitive plasma treatment [21]. Similar findings were made in volunteers with plasma-treated, laser-induced wounds in a 1-year follow up study [24]. Moreover, a significant increase in oxidative DNA products was not found, which would be representative of excessive plasma-induced DNA damage being potentially mutagenic. For this, 8-hydroxy-2′—deoxyguanosine (8-OHdG) was quantified, being one of the predominant forms of ROS-induced oxidative DNA alteration [55]. Although several reports have speculated DNA-damaging properties of plasma treatment [56,57,58,59,60], these reports usually did not use gold-standard assays of genotoxic research or only partially adhered to them to confirm their results. Our previous reports did not find, for instance, an increase in micronucleus formation following plasma treatment [20,35,61], being a gold standard assay, according to the OECD (Organisation for Economic Co-operation and Development) for toxicity research. A lack of genotoxicity was also obtained using the HPRT (Hypoxanthin-Guanin-Phosphoribosyltransferase) test [22], another OECD accredited assay. Analyzing the sub-G1 fraction during cell cycle analysis, as done by some studies, is not an indicator of DNA damage but a regular event during the apoptotic cascade, where DNA is cleaved into smaller pieces to avoid excessive inflammation. Hence, the appearance of sub-G1 cells in the cell cycle analysis takes place for any toxic stimulus, regardless of its DNA-damaging nature. Additionally, many reports use the presence of phosphorylated histone 2AX (γH2AX) as a marker for DNA double-strand breaks. While this seems intuitive, since γH2AX is a frequently used marker in radiobiology [62], we recently discovered that following plasma treatment with γH2AX is a consequence of apoptosis rather than its cause due to genotoxic events [35]. Therefore, and in line with previous reports in vitro, in vivo, and in patients, plasma treatment indisputably seems to be safe in our model in terms of pathological transformation in vivo. Moreover, this work mounts direct evidence that the action of plasma treatment is mainly localized to the site of treatment and does not affect the systemic redox status.

Our results confirmed the stimulating properties of plasma treatment in the skin that possibly contribute to the promotion of wound healing. This links to previous results with the kINPen treatment of wounds where, similar to our findings, an increase in cell proliferation and expression of Nrf2 and catalase was observed [36]. An increase in cell proliferation following plasma treatment was also previously observed in vitro in human HaCaT keratinocytes [63,64]. Ex vivo human skin exposed to plasma confirmed such an increase of proliferation in keratinocytes, while an increase of γH2AX was not found [65]. Such a dual role of plasma treatment being stimulating or toxic, depending on the state of the cells or tissues being targeted, was previously noted already [26,28]. Nrf2 is a potential biomarker of head and neck SCC and its expression correlates with disease appearance [66]. Plasma treatment reduced Nrf2 levels in our murine model of UVB-induced skin lesions, suggesting the involvement of Nrf2 in SCC pathogenesis [67]. In general, it is noteworthy that, in our study, plasma treatment increased Nrf2 levels in normal skin while reducing Nrf2 levels in UVB-induced skin lesions. It is a regularly observed phenomenon in biology that a silent pathway is prone to activation induced by an agent, while an overactive pathway receiving additional stimuli experiences negative feedback regulation, ultimately resulting in its downregulation, as seen, for example, for nitric oxide in macrophages [68]. For Nrf2, negative feedback regulation is also described through interaction with KEAP1 (Kelch-like ECH-associated protein 1) and ATF3 (Activating Transcription Factor 3) [69], as well as GSK-3β (Glycogen Synthase Kinase 3 Beta) [70], mechanisms possibly triggered by plasma treatment.

The selective toxicity of plasma treatment in vitro was demonstrated since human malignant keratinocytes (SCC) were significantly more sensitive to plasma treatment compared to human non-malignant (HaCaT) keratinocytes. This is in line with previous reports, showing a cytotoxic action and self-amplification of mitochondrial ROS production, following treatment in various human SCC cell lines, including FaDu, SNU1041, OSC19, SNU800, and HN9 [47,71,72]. Another study reported the involvement of catalytic iron in eliciting ferroptosis in plasma-treated human and mouse SCC lines, such as SAS, Ca9-22, HSC-2, HSC-3, HSC-4, Sa3, and Ho-1-u-1 [73]. This and another work [29] also suggested a certain degree of selectivity of plasma treatment towards cancer cells. This might be based on differences in the signaling responses translating a pro-oxidant milieu into cell death. For plasma medicine, molecules involved in this process might be related to, for instance, the Nrf2 pathway [36,74,75], heme oxygenase 1 (HMOX1) [76], or other antioxidant response elements [77]. Others have suggested that the lipid composition of tumor cells [78,79,80] and the expression of aquaporins [81,82,83] are also important for the selectivity of plasmas towards malignant cells. Plasma generates different ROS, such as ozone (O_3_), superoxide (O_2_^−^), singlet delta oxygen (^1^O_2_), atomic oxygen (O), hydroxyl radical (^•^OH), and hydrogen peroxide (H_2_O_2_) [84,85,86,87,88,89,90,91,92,93]. A study comparing different plasma feed gas compositions suggested thar nitrogen plasma inhibits SCC growth and migration most potently [94], which is in line with another report, which found nitric oxide (NO) to be important [95]. The kINPen plasma jet used in our study generates both reactive nitrogen species and NO, among other species [96]. This jet also generates a number of other types of reactive species, as thoroughly outlined in a recent review [97]. Which of these species are predominant in mediating effects in vivo is currently a topic of investigation, and conclusions are limited by the types of analysis available in distinguishing several types of reactive species from each other. For the in vitro results, the dominant types of ROS mediating an effect are long-lived oxidants, such as H_2_O_2_, and possibly nitrite and nitrate [98,99]. However, in vivo, other plasma agents may have contributed to the effects observed. The kINPen generates UV radiation but only to a low amount (1/30 of the allowed radiation) [100], at least in the non-conductive mode. Moreover, the UV treatment, but not the plasma treatment, led to SCC lesions in our animal model, exemplifying that the biologically relevant UV emission of the kINPen is not pathological. Our plasma jet also generates thermal radiation, but this is in the range of body temperature, making a contribution of heat to the effects of plasma observed unlikely. Another attribute of plasma is the presence of electric fields. However, argon plasma jets, such as the kINPen, have significantly lower electric field intensities compared to helium plasma jets or dielectric barrier discharges. This is exemplified by the fact that the electric field of the kINPen is insufficient to induce immediate permeabilization of the cell membrane [101]. The possibility of electrons and ions generated by the plasma contributing to the effects observed in vivo cannot be excluded.

An underexplored research avenue is the combination of plasma treatment with existing therapeutic regimens to potentiate cytotoxic effects against SCC. Already in 2009, a proof-of-concept study suggested additive cytotoxicity of antibody-conjugated gold nanoparticles with plasma treatment against human YD-9 SCC cells in vitro based on results they have obtained with melanoma cells [102]. Promising results were seen in the combination of plasma therapy with traditional agents in melanoma treatment [103,104,105], which warrants further exploration for research on SCC in the future. This might also relate to combining plasma therapy with other physical modalities, such as pulsed electric fields, as recent reports have shown additive toxicity of both treatments in vitro [101,106].

## 4. Methods

### 4.1. Cell Culture

The in vitro experiments were carried out using three human cell lines, namely A431 (ATCC: CRL-1555) and SCC13 (CVCL-4029), as malignant cutaneous squamous cell carcinoma cell lines, and non-malignant HaCaT keratinocytes. Cell culture was performed in Dulbecco′s modified Eagle′s medium (DMEM; Pan Biotech, Aidenbach, Germany), supplemented with 10% fetal calf bovine serum (FCS), 2% glutamine, and 1% penicillin and streptomycin (Sigma, Steinheim, Germany). For experiments, absolute cell counting was performed in a semi-automatic manner using acoustic focusing flow cytometry (Thermo Scientific, Waltham, USA). For 2D experiments, cells were resuspended in Roswell Park Memorial Institute 1640 (RPMI; Pan Biotech, Aidenbach, Germany) medium at 1.25 × 10^5^ per ml, and 750 µL were added per well of a 24-well plate (Eppendorf, Hamburg, Germany; catalog number: 0030722116). The plates have a rim that can be filled with sterile water to protect the cultures from excessive evaporation and the ′edge effect′. For 3D tumor spheroid formation, 1 × 10^4^ cells were seeded in ultra-low attachment plates (Nunclon Sphera; Thermo Scientific, Waltham, MA, USA) and centrifuged.

### 4.2. Plasma Treatment and Cell Analysis

Plasma treatment was done using high-purity (99.999%) argon gas (Air Liquide, Paris, France) at two standard liters per minute to ignite the atmospheric pressure argon plasma jet kINPen (neoplas tools, Greifswald, Germany). The jet is well investigated and its operation and output parameters have been described in detail before [97]. The plasma treatment was performed in a standardized manner using an *xyz*-table (CNC Step, Gelfern, Germany), which fixed the plasma jet (distance from the nozzle to the liquid was 20 mm and kept constant) and operated the computer-driven treatment protocol. The support on which the tests are performed (e.g., conductive or dielectric surface) can influence the plasma characteristics and, therefore, the treatment [107]. The plasma treatment was done with the jet in non-conductive mode, hovering over a well plate of liquid containing the cells. The well plate was located on a plastic holder, which in turn was fixed on a grounded metal plate. Several plasma treatment times were employed. Evaporation was compensated by adding a pre-determined amount of double-distilled water. To analyze cytotoxic plasma effects in the 2D cultured cells in vitro, 1 × 10^4^ cells were added in complete medium and allowed to adhere. After plasma treatment, resazurin (100 µM; Alfa Aesar, Haverhill, MA, USA) was added 20 h later. After several hours of incubation, the dye was transformed via reduction reactions in metabolically active cells to the fluorescent resorufin. Fluorescence was determined using a multiplate reader (Tecan, Männedorf, Switzerland) at λ_ex_ 535 nm and λ_em_ 590 nm and normalized to that of untreated control cells. Tumor spheroids were generated by adding 1 × 10^4^ cells to ultra-low attachment plates (Nunc, Roskilde, Denmark), followed by the centrifugation of the plates at 1000× *g* and further incubation. To analyze cytotoxic plasma effects in the 3D cultured cells in vitro, sytox blue (1 µM; Thermo Scientific, Waltham, USA) was added to the wells 24 h post-exposure. Sytox blue is a nuclear and chromosome counterstain. Because it is impermeant to live cells, it can be used as an indicator of dead cells within a population. The spheroids were subjected to high content imaging analysis (Operetta CLS; PerkinElmer, Hamburg, Germany) using a 5x air objective (NA = 0.16; Zeiss, Jena, Germany) to capture brightfield (BF) and sytox blue (SB) fluorescence at λ_ex_ 365 nm and λ_em_ 430–500 nm. Data analysis of the sum intensity of sytox blue (SB) was done using Harmony 4.9 Software (PerkinElmer, Hamburg, Germany).

### 4.3. Generation of UVB-Induced, SCC-Like Skin Lesions In Vivo

Female 20-weeks-old HRS/J hairless mice weighing between 20 to 25 g were obtained from the central animal facility of the State University of São Paulo (USP). The animals were provided with access to water and food ad libitum, and handling was in accordance with the National Institution of Health guidelines for the welfare of experimental animals. The study was approved by the Research Ethics Committee (approval number 11633.2019.88) of the State University of Londrina, Paraná State, Brazil. To induce skin transformation, animals were exposed to UV irradiation, using the fluorescent lamp PHILIPSTL/12 40W UVB, which emits radiation from 270 to 400 nm with a maximum peak at around 313 nm. The lamp was embedded in a 1.30 m × 0.43 m × 0.45 m box, where the mice were placed in cages 15 cm beneath the lamp. The boxes contained apparatus to ensure that the irradiation was directed toward the dorsal skin while simultaneously allowing for free movement of the animals. UVB output was measured using a Research Radiometer model IL-1700 (International Light, USA) with a radiometer sensor for UV (SED005) and UVB (SED240). A total of 73% of the total UV irradiation under the experimental conditions was UVB. For the experiments, the animals were separated into four groups with five animals each: untreated (non-irradiated control), plasma alone, UVB alone, and UVB + plasma. The UVB irradiation (Figure 2A) started in the initiation phase, where mice from the UVB groups (Figure 2B) were exposed to 0.228 J/cm^2^ of UV for 16 min, 5 days a week, for 2 weeks. The animals then rested for 1 week, followed by a promotion phase, where they were irradiated with UVB every other day up to week 11. On average, there were 1–3 lesions per animal. The kINPen plasma treatment started at the eighth week with 3 min treatment time per lesion every other day (alternating with UVB treatment). The animals in the plasma group (without UVB pre-treatment) received the same plasma treatment (Figure 2C). During the plasma treatment, the animals were anesthetized with ketamine/xylazine. This was not done because the plasma treatment was painful [14] but to reduce the stress the animals would have experienced during the prolonged immobilization period. Plasma treatment was performed on a heated and grounded surface. At the end of the 11th week, the animals were euthanized by cervical dislocation while still under anesthesia.

### 4.4. Sample Collection

Heparinized blood was collected by cardiac puncture and centrifuged at 1100× *g* to obtain blood plasma and erythrocytes. Blood plasma was stored at −20 °C. Erythrocytes were washed three times with 0.9% NaCl and maintained at 4 °C in Alsever′s solution. Cutaneous UVB-induced skin lesions were removed from the dorsal skin of UVB irradiated animals for histopathological and immunohistochemical analysis. Lesion-free, adjacent dorsal skin samples were removed and stored at −80 °C for total radical trapping antioxidant parameter (TRAP) assay. An equivalent area of tissue was collected in the control and plasma groups.

### 4.5. Histopathological and Immunohistochemical Analysis

Cutaneous lesions were fixed in 10% neutral buffered-formalin, stored in 70% ethanol, and embedded in paraffin before sectioning. Sections of 5 µm were cut and stained with hematoxylin and eosin (H&E). The histopathological evaluation was made in a blinded study considering characteristics of transformed skin, such as epidermal thickness, follicular atrophy, keratin pearls, and the presence of atypical keratinocytes proliferating into the dermis [5]. For immunohistochemistry, 5 μm sections were mounted on silanized slides, deparaffinized, rehydrated, immersed in 15 mmol/L citrate buffer (pH 6.0), and subjected to heat-induced epitope retrieval using a vapor lock for 40 min. The slides were rinsed with phosphate-buffered saline (PBS) and immersed in 3% hydrogen peroxide for 30 min to block endogenous peroxidases. Non-specific protein binding was blocked with 3% BSA (bovine serum albumin) solution for 30 min. The sections were then incubated with primary monoclonal antibodies targeted against catalase (1:50; Santa Cruz, Dallas, TX, USA), Ki-67 (1:100; Santa Cruz, Dallas, TX, USA), Nrf2 (1:250; Santa Cruz, Dallas, TX, USA), or 8-OHdG (1:100; Santa Cruz, Dallas, TX, USA) for 2 h at 37 °C in a humid chamber. After incubation and washing, peroxidase-conjugated secondary anti-rabbit IgG (Sigma, Steinheim, Germany) was added for 1 h at room temperature, followed by their chromogenic detection, using 3,3′-diaminobenzidine (DAB; Sigma, Steinheim, Germany). The slides were counterstained with Harris′s hematoxylin, dehydrated, and mounted with Permount (Biomeda, Foster City, CA, USA). Negative controls were done by omitting the primary antibody. The intensities of immunoreactivities against the primary antibody used were examined using a photomicroscope (Olympus BX41, Olympus Optical, Hamburg, Germany). For image analysis via ImageJ [108], the percentage of DAB staining was quantified in three fields of six different slides for each group. The average of the three fields was considered.

### 4.6. Analysis of Local and Systemic Oxidative Stress Parameters

Irradiated dorsal lesion-free skin samples from mice were defrosted and homogenized using an Ultra-Turrax homogenizer, containing 10 mg/mL or 50 mg/mL of tissue in buffer and centrifuged (11,000× *g* for 15 min at 4 °C). The 50 mg/mL supernatant was used for measurement of catalase activity [109] and reduced glutathione (GSH) content [110], while the 10 mg/mL supernatant was used to determine the total radical trapping antioxidant parameter (TRAP), as described before [111]. To analyze systemic GSH [110] and superoxide dismutase (SOD) activity [112], erythrocytes were washed and diluted in deionized water at ratios of 1:80 and 1:20, respectively. Blood plasma samples were defrosted and diluted at 1:2 for malondialdehyde (MDA) quantification via HPLC, indicating lipid peroxidation [113]. For normalization purposes, the total protein content was measured in erythrocytes (1:80) and tissue homogenate (50 mg/mL) [114,115].

### 4.7. Statistical Analysis

Graphing and statistical analysis were performed using prism 8.4.2 (GraphPad Software, San Diego, CA, USA). Mean and standard errors were calculated and graphed and statistical analysis was performed using an unpaired t-test, as indicated, or one-way or two-way analysis of variances (ANOVA), as indicated.

## 5. Conclusions

The aim was to test cold physical plasma as a means to target malignant lesions of the skin therapeutically. This was motivated by previous findings, suggesting an anticancer activity of plasma technology. Plasma treatment selectively limited carcinogenic progression of squamous cell carcinoma in vitro and in vivo via reduction of cell proliferation and modulation of redox balance. Moreover, it was identified that plasma treatment acted locally but not systemically, at least in terms of oxidative and antioxidative parameters that are tightly linked to the primary biological mechanism of plasma systems, reactive oxygen, and nitrogen species. Furthermore, reducing the harmful effects of existing SCC therapies may be achieved by combining plasma treatment with existing SCC therapies to enhance their clinical efficacy, an increasingly important approach in oncology.
